# The SDF-1α/CXCR4 Axis is Required for Proliferation and Maturation of Human Fetal Pancreatic Endocrine Progenitor Cells

**DOI:** 10.1371/journal.pone.0038721

**Published:** 2012-06-22

**Authors:** Ayse G. Kayali, Ana D. Lopez, Ergeng Hao, Andrew Hinton, Alberto Hayek, Charles C. King

**Affiliations:** Department of Pediatrics, Pediatric Diabetes Research Center, University of California San Diego, San Diego, California, United States of America; University of Bremen, Germany

## Abstract

The chemokine receptor CXCR4 and ligand SDF-1α are expressed in fetal and adult mouse islets. Neutralization of CXCR4 has previously been shown to diminish ductal cell proliferation and increase apoptosis in the IFNγ transgenic mouse model in which the adult mouse pancreas displays islet regeneration. Here, we demonstrate that CXCR4 and SDF-1α are expressed in the human fetal pancreas and that during early gestation, CXCR4 colocalizes with neurogenin 3 (ngn3), a key transcription factor for endocrine specification in the pancreas. Treatment of islet like clusters (ICCs) derived from human fetal pancreas with SDF-1α resulted in increased proliferation of epithelial cells in ICCs without a concomitant increase in total insulin expression. Exposure of ICCs *in vitro* to AMD3100, a pharmacological inhibitor of CXCR4, did not alter expression of endocrine hormones insulin and glucagon, or the pancreatic endocrine transcription factors PDX1, Nkx6.1, Ngn3 and PAX4. However, a strong inhibition of β cell genesis was observed when *in vitro* AMD3100 treatment of ICCs was followed by two weeks of *in vivo* treatment with AMD3100 after ICC transplantation into mice. Analysis of the grafts for human C-peptide found that inhibition of CXCR4 activity profoundly inhibits islet development. Subsequently, a model pancreatic epithelial cell system (CFPAC-1) was employed to study the signals that regulate proliferation and apoptosis by the SDF-1α/CXCR4 axis. From a selected panel of inhibitors tested, both the PI 3-kinase and MAPK pathways were identified as critical regulators of CFPAC-1 proliferation. SDF-1α stimulated Akt phosphorylation, but failed to increase phosphorylation of Erk above the high basal levels observed. Taken together, these results indicate that SDF-1α/CXCR4 axis plays a critical regulatory role in the genesis of human islets.

## Introduction

The need to find β-cell sources independent of human cadaveric sources useful for the development of cell-based therapies for patients with type 1 diabetes depends to a great extent on enhanced understanding of the molecular mechanisms that regulate human endocrine pancreas maturation. These insights will help the derivation of new protocols for both differentiation of human embryonic stem cells (hESCs) and regeneration of the compromised endocrine pancreas either from sources such as acinar tissue, other endocrine hormone expressing cells, or the remaining β-cells.

Chemokines are a superfamily of small secreted (8–10 kD) cytokines that bind and activate heptahelical transmembrane G-protein coupled receptors (reviewed in [1]) that are involved in a number of diverse biological processes, including leukocyte trafficking [2], [3], regulation of HIV infection [4], mobilization of hematopoietic stem cells [5], regulation of angiogenesis [6], metastasis and fetal development [7]. Although a number of chemokines play critical roles in organogenesis [8], SDF-1α and CXCR4 comprise the only chemokine/chemokine receptor pair that individually results in embryonic lethality in mouse knock-outs. Mice with genetic disruption of either the CXCR4 receptor or SDF-1α ligand display abnormal gastrointestinal vasculature, aberrant migration of cerebellar neurons, impaired B-lymphopoiesis, cardiac ventricular septal defects, and failure of bone marrow hematopietic colonization [9], [10], [11], [12]. Identical phenotypes of the knockouts for SDF-1α and CXCR4 suggest that CXCR4 is the only receptor for SDF-1α, although recent studies have demonstrated that SDF-1α can also bind and activate CXCR7 [13].

The recent finding that CXCR4 is a marker for definitive endoderm (DE) during the differentiation of human embryonic stem cells (hESCs) led us to investigate the fate of this receptor between DE formation and the generation of hormone producing endocrine cells. While the mechanism of action of CXCR4 in this context has not been studied, we have previously documented SDF-1α/CXCR4 receptor pair expression in fetal mouse pancreas and its obligatory function in an adult mouse model of pancreatic regeneration [14]. In these transgenic mice in which IFNγ is expressed under the control of the insulin promoter, the pancreas displays ductal proliferation and islets exhibit regeneration [15], [16], [17], [18]. In this system, SDF-1α stimulated migration and activation of the signaling molecules MAPK, Akt, and Src in pancreatic ductal cells. A protective effect on ductal cell apoptosis and a parallel induction of ductal proliferation was observed *in vivo* [14], [18]. Other studies have also explored the role of CXCR4 in development. In zebrafish lacking CXCR4 receptor, duplication of endodermal organs including liver and pancreas was observed [19]. Additionally, in a study of the second stage epithelial transition in embryos from SDF-1α and CXCR4 knockout mice, Hick et al reported transient defective morphogenensis in the ventral and dorsal pancreas, suggesting an important role of this pathway in the branching morphogenesis of endocrine pancreas development [20].

In the present study, we identify the SDF-1α/CXCR4 signaling axis as an important component of human fetal β-cell development and begin to uncover the downstream signaling events that are critical for this process. Using Immunofluorescence, CXCR4 expression is tracked through human fetal pancreas development and demonstrated to exclusively co-localize with insulin positive cells during later stages of development. Furthermore, CXCR4 activity is demonstrated to be essential for the *in vivo* differentiation of islet-like clusters into β-cells and that SDF-1α is required for the proliferation of epithelial endocrine precursors through activation of PI 3-kinase and Akt. Taken together, these data identify SDF-1α/CXCR4 signaling as a critical component of islet genesis.

## Results

### Localization of CXCR4 Expression in Human Fetal and Adult Pancreas

**Figure 1:**
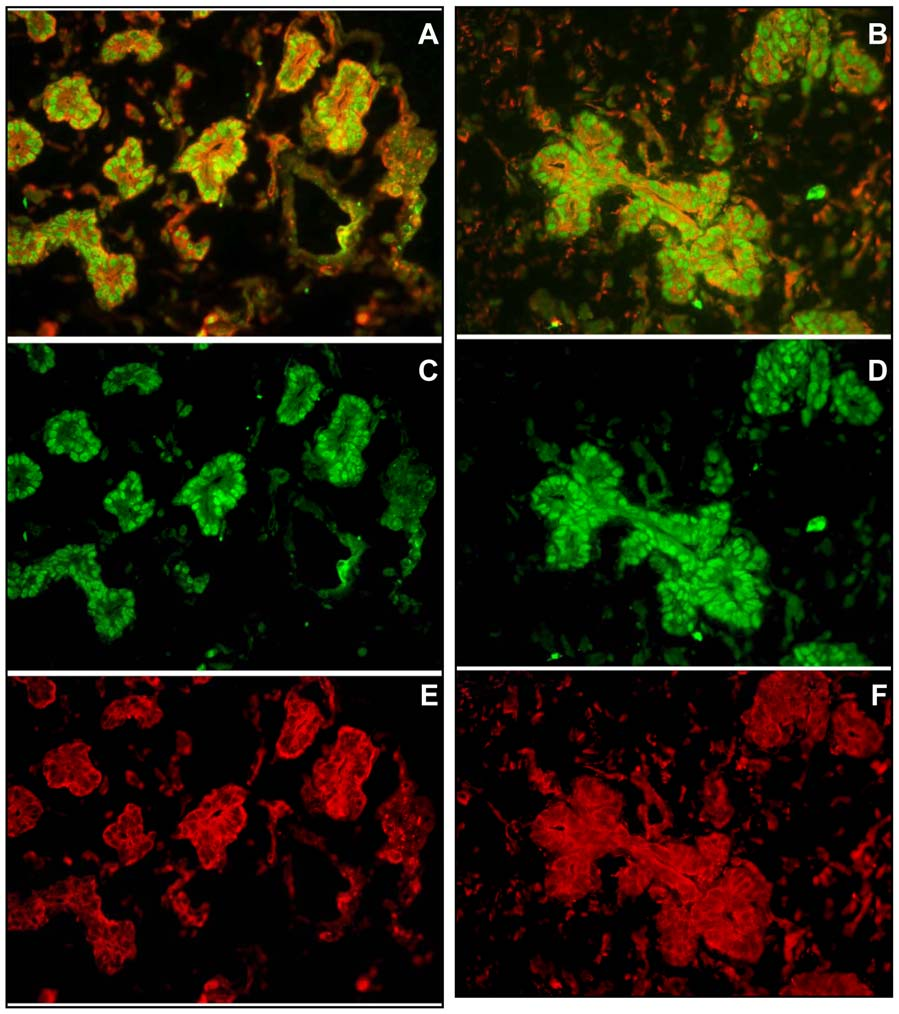
CXCR4 and Ngn3 are co-expressed in the branching epithelia of 11-week gestational human fetal pancreas. Photomicrographs (20X) of two representative areas depict Ngn3 (green) in nuclei and CXCR4 (red) in membranes. The composite images (A, B) are resolved into their green (C, D) and red (E, F) channels for optimal visualization.

Our laboratory and others had previously identified SDF-1α/CXCR4 expression and signaling in mouse islets [14], [21]. Given that the CXCR4 receptor is also used as a marker of definitive endoderm in human embryonic stem cells [22], we performed immunofluorescence to explore the relationship between CXCR4 expression and endocrine specification. In 11.6-week human fetal pancreas, cells expressing CXCR4 also expressed neurogenin 3 (ngn3), a transcription factor necessary for endocrine commitment (Fig. 1). Therefore, in the epithelial migration in the early stages of formation of islet-like clusters in the human pancreas, the ngn3 positive cells that are destined to differentiate into endocrine cells are all marked by CXCR4.

**Figure 2:**
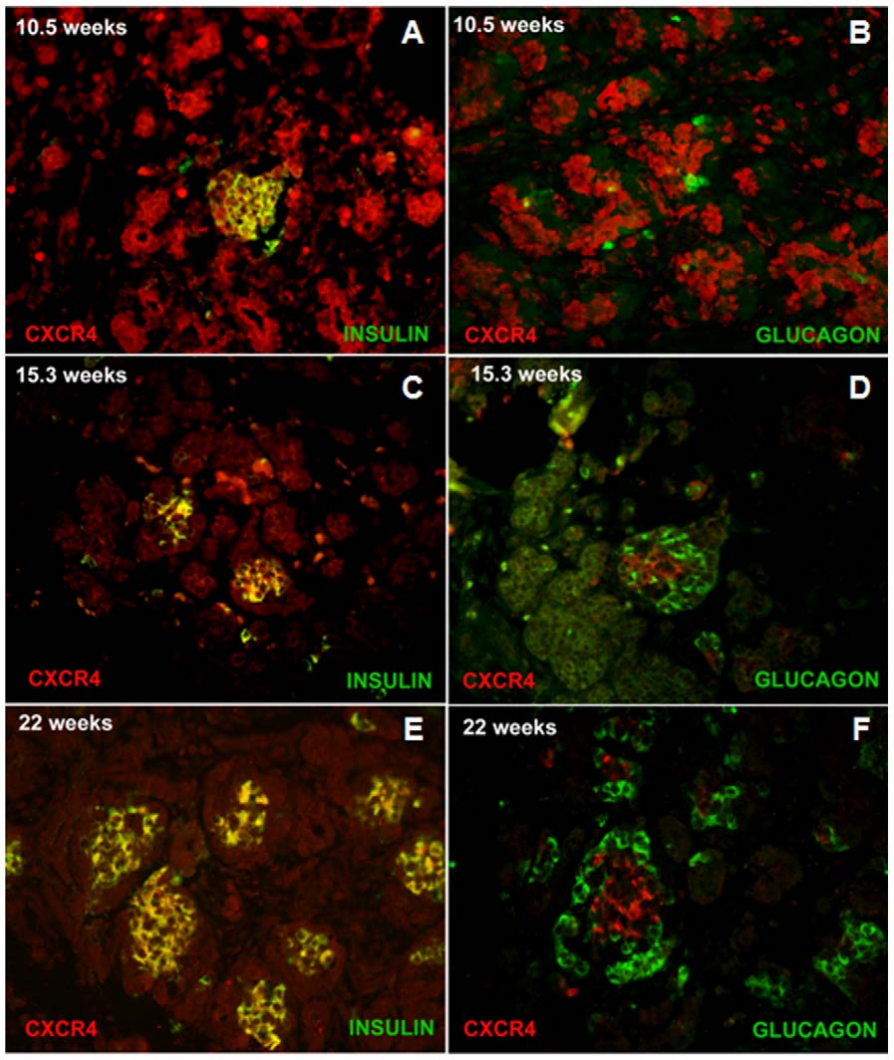
Time course of CXCR4, insulin, and glucagon expression in human fetal pancreas. At 10.5 weeks of gestation CXCR4 (red) is expressed throughout the pancreatic epithelia (A and B). Most of the insulin-expressing cells (green) in islet-like structure appear yellow because they co-express CXCR4 (A). B. The few glucagon (green) expressing cells at 10.5 weeks of gestation do not express CXCR4. At 15.3 weeks of gestation CXCR4 expression is restricted to the islet-like clusters, which also express insulin (green), again appearing yellow C. The glucagon (green) expressing cells in the islet like structures are contiguous to the CXCR4 expressing cells and no co-expression is detected D. By 22 weeks, the glucagon (green) positive cells, also located in the islet-like cluster, surround the CXCR4 (red) positive cells (F), which co-express with insulin.

**Figure 3:**
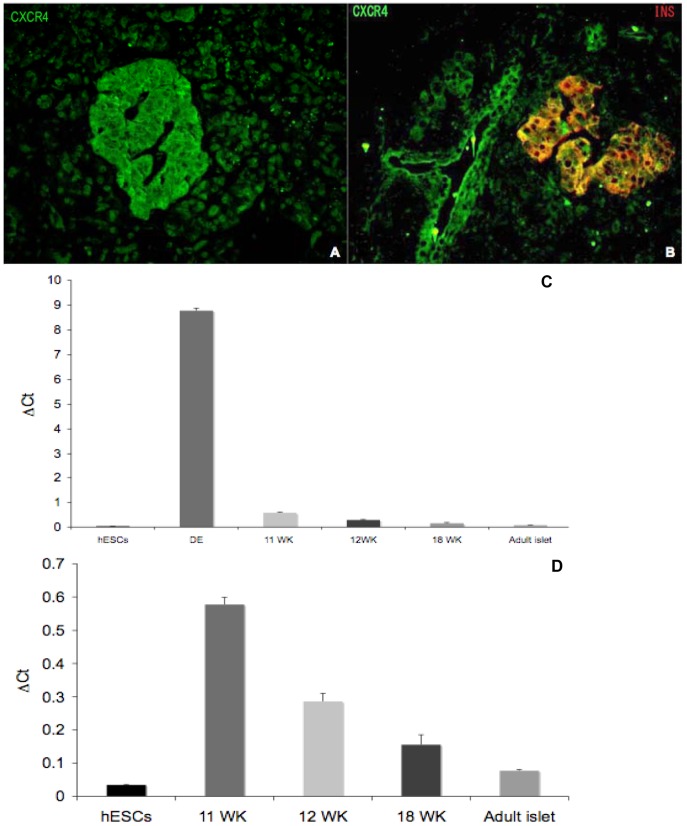
CXCR4 is expressed in human adult islets and ducts. Panel A illustrates CXCR4 (green) expression in human adult islets; note the absence of CXCR4 staining in exocrine tissue surrounding the islet. Panel B reveals that in the human adult islet CXCR4 expressing cells coexpress insulin (yellow). Human adult ducts also exhibit CXCR4 (green) staining. C. RT-qPCR analysis of CXCR4 mRNA in human embryonic stem cells (hESCs), hESC derived definitive endoderm (DE), fetal islet cell clusters at 11, 12 and 18 weeks gestation (11 WK, 12 WK, 18 WK), and adult islets. Expression levels expressed as ΔCt values relative to Cyclophilin G. D. Due to extremely high expression in DE, CXCR4 mRNA levels shown comparing only hESCs, fetal ICCs, and adult islets.

Using immunofluorescence microscopy, we next explored CXCR4 expression in human fetal islets at various stages of development (Fig. 2). At week 10.5 of gestation, CXCR4 was diffusely expressed throughout the pancreatic epithelium, including co-expression with the few cells that were insulin positive (Fig. 2A). Glucagon-positive cells, however, did not express CXCR4 at the same gestational age (Fig. 2B). In islet-like clusters (ICCs), at both 15.3 and 22 weeks of gestation, CXCR4 expression became restricted primarily to insulin (Fig. 2C and E), but not glucagon positive cells (Fig. 2D and F). Furthermore, CXCR4 was expressed both in the islets and the ducts of human adult pancreas, but not in acinar tissue (Fig. 3A and B). CXCR4 mRNA levels in all ICC samples were much lower than CXCR4 levels found in definitive endoderm, the first committed stage of endocrine cell development characterized by very high CXCR4 levels (Fig. 3C). However, compared to pluripotent human embryonic stem cells, the four different human fetal pancreatic samples and human adult islets displayed significantly higher CXCR4 expression (Fig. 3D).

**Figure 4:**
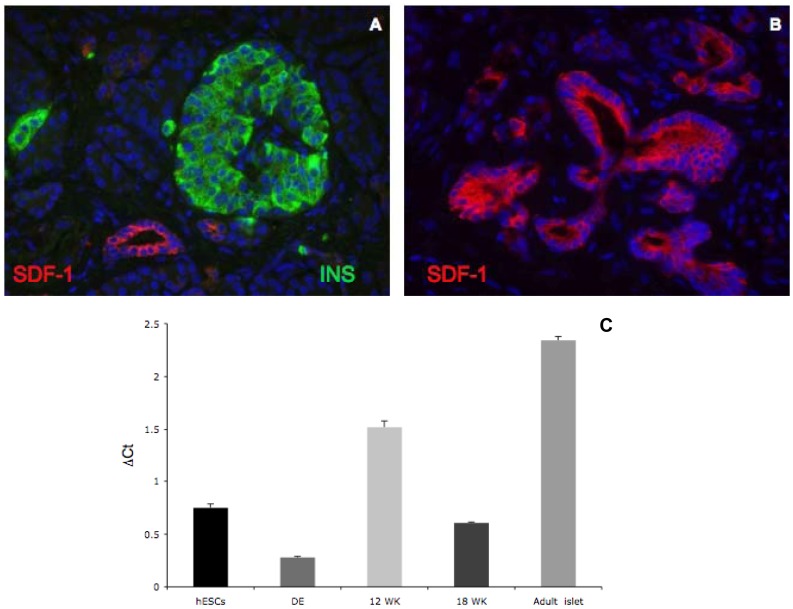
SDF-1 expression in human adult pancreas. Panels A and B. Double immunofluorescent staining of SDF-1α (red) and insulin (green) in human adult islets demonstrates ductal enrichment of SDF-1α, but no expression in islets. Panel C. SDF-1α mRNA expression in hESCs, definitive endoderm (DE), 12 week (12 WK) and 18 week (18 WK) human fetal ICCs and adult islets. mRNA levels are expressed as ΔCt values relative to Cyclophilin G.

We next undertook studies to determine whether expression of SDF-1α, the CXCR4 ligand, could be detected in human adult pancreas. Expression of SDF-1α was restricted to the ducts of the human adult pancreas (Fig. 4A–B). RT-PCR analysis showed that SDF-1α is expressed in heterogeneous cell populations of human fetal and adult islet samples, as well as pluripotent human embryonic stem cells and definitive endoderm (Fig. 4C). Taken together, these data reveal that CXCR4 is expressed in widely in human fetal pancreatic endoderm, but later in development is selectively expressed in ductal tissue and insulin expressing cells in the human fetal and adult pancreas; whereas SDF-1α is expressed and localized to ducts in human adult pancreas.

### Effect of SDF-1α/CXCR4 Axis on β Cell Maturation

#### *In vitro* study

**Figure 5:**
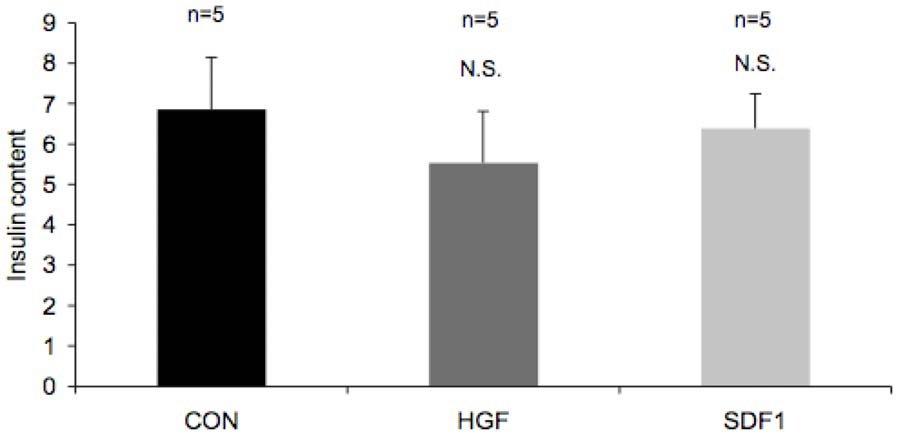
HGF or SDF-1 treatment of human ICCs *in vitro* does not alter insulin content. Insulin content expressed as picomoles/µg DNA of fetal ICCs after 5-day culture in medium containing 10% human serum or serum plus HGF or SDF-1α was assessed. Data represents the average of five separate sets of experiments. Values are expressed as mean ± SEM and were not significant by Student’s t-test.

Human fetal ICCs can be maintained in suspension *in vitro* for up to 5 days without compromising their integrity [23]. Previous studies from our group have shown that *in vitro* treatment of ICCs with Exendin 4 [24] and keratinocyte growth factor (KGF) [25] followed by transplantation under the kidney capsule of immunocompromised mice and continued *in vivo* Exendin 4 or KGF treatment resulted in accelerated maturation and proliferation of β-cells. We first wanted to determine whether treatment of the ICCs with SDF-1α would accelerate *in vitro* differentiation of the β-cells. ICCs derived from human fetal islets (12 to 16.5 week gestational-age) were treated with SDF-1α (100 ng/ml) or hepatocyte growth factor (HGF; 10 ng/ml) *in vitro* every other day for a period of five days. HGF is a growth factor that is the primary component of the mesenchyme induced β-cell growth in fetal ICCs [26]. *In vitro* treatment with SDF-1α or HGF did not result in a significant change in insulin content as measured by ELISA (Fig. 5).

**Figure 6:**
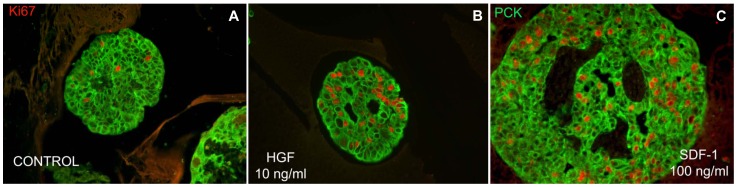
SDF-1 stimulates proliferation of epithelial cells in ICCs from early gestational human fetal pancreas. ICCs isolated from fetal pancreas of 12 to 14 weeks were grown in suspension culture, treated with 10% human serum alone, with HGF (10 ng/ml) or with SDF-1α (100 ng/ml) for six days. ICCs were fixed and analyzed by immunofluorescence staining. PanCytokeratin (green) is an epithelial marker. Ki67 (red) is used as a marker of proliferation.

In these early gestation ICCs, incubation with human serum was sufficient to induce epithelial cell proliferation (Fig. 6A). Treatment with HGF or SDF-1α further stimulated the proliferation of epithelial cells (Fig. 6B and C, respectively). SDF-1α was as efficient as HGF at inducing cell proliferation, indicating a role for this receptor in cell growth and development. These data suggest that during the earlier stages of human pancreatic β-cell development, both HGF and SDF-1α can act as potent mitogens.

**Figure 7:**
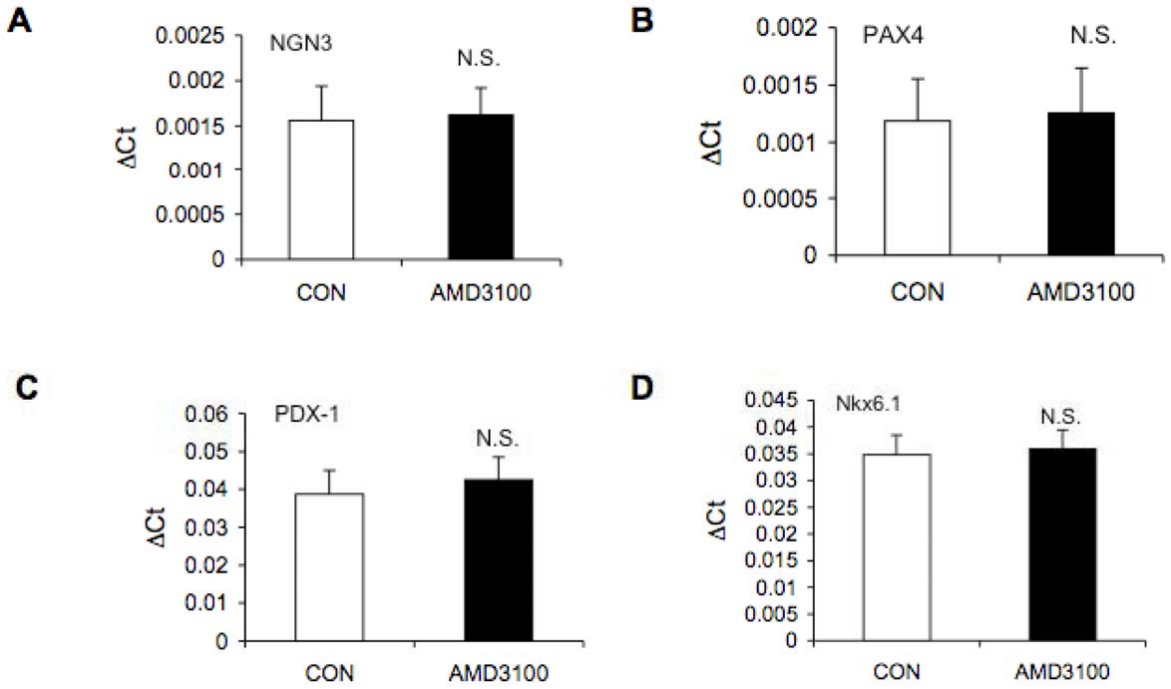
Comparison of mRNA levels for endocrine markers in control and AMD3100 treated ICCs derived from human fetal pancreas by RT-PCR. The values of mRNA for endocrine markers are expressed as mean ± SEM of ΔCTs of the transcription factors compared to Cyclophilin A. Ngn3 (Panel A), PAX-4 (Panel B) and MAF-A (data not shown) were detected at approximately 35 to 37 PCR cycles, PDX-1 (Panel C) and Nkx6.1 (Panel D) were detected at 30 to 31 PCR cycles both in control and AMD3100 treated ICCs. Four different preps from gestational ages 13 to 17 weeks were analyzed. N.S.  =  not significant by Student’s t-test.

To determine whether SDF-1α expression in ICCs regulates proliferation or maturation in a paracrine manner, we next asked whether pharmacologic inhibition of CXCR4 by AMD3100 could impact the expression of transcription factors important in pancreatic endocrine differentiation. RT-PCR analysis of PDX-1, ngn3, Pax4, MAFA, Nkx6.1, insulin, and glucagon was performed in RNA from control and AMD3100 treated ICCs from 13 and 17 week-gestational human fetal pancreata (Fig. 7). There was no significant difference in expression of any of the transcription factors measured upon treatment with AMD3100. Insulin and glucagon expression increased with gestation but did not change with AMD3100 treatment (data not shown).

#### *In vivo* study

**Figure 8:**
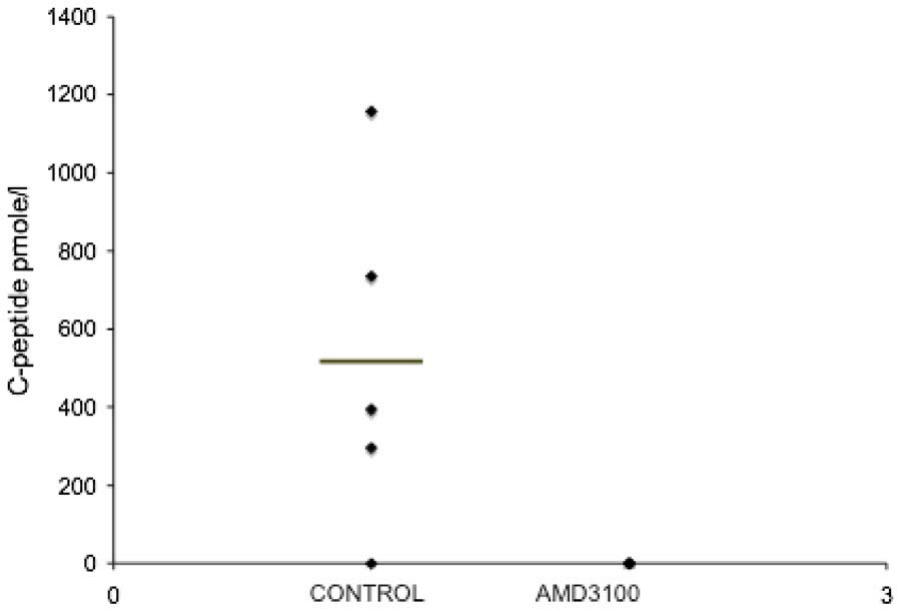
Serum Human C-peptide levels in nu/nu mice transplanted with human fetal ICCs treated with AMD3100. Sixteen weeks after transplantation, circulating human C-peptide levels were measured 30 minutes after a glucose challenge in fasted nu/nu mice that had been treated with saline or AMD3100. For both the saline and AMD3100 groups n = 5. For the control group, C-peptide levels were 517.3±198.83 (mean±SEM). C-peptide levels in the AMD3100 treatment group were undetectable. P<0.05 by Student’s t-test.

**Figure 9:**
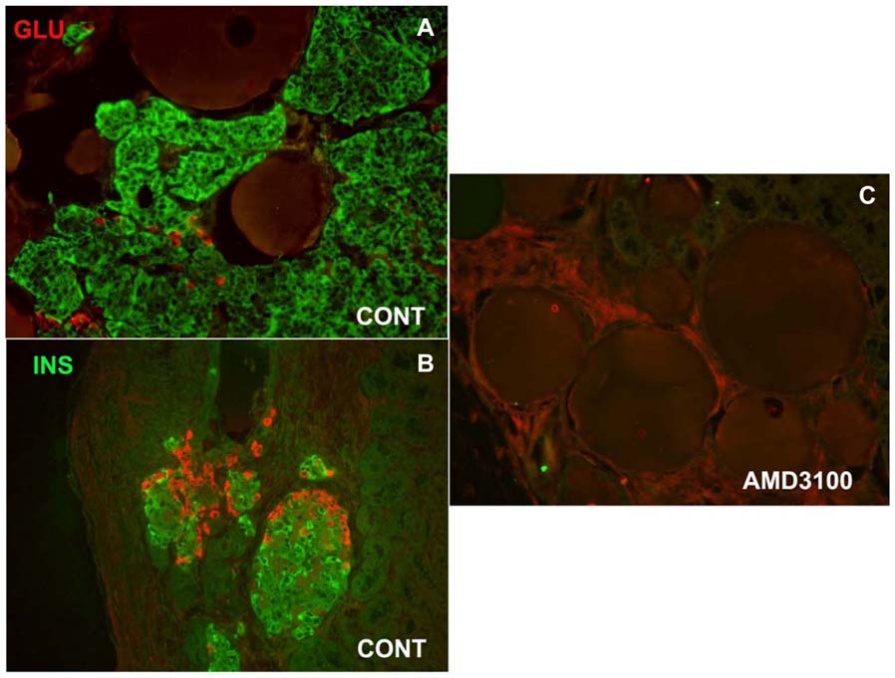
Expression of insulin and glucagon in grafts from control and AMD3100 treated ICCs transplanted into nu/nu mice. Double immunofluorescent staining of insulin (green) and glucagon (red) in ICCs transplanted under the kidney capsule of nu/nu athymic mice treated with saline (A, B) or AMD3100 every two days during two weeks following transplantation. Insulin and glucagon expression is extensive in the control ICCs. C. Insulin or glucagon was not detectable in the grafts from the mice treated with AMD3100. Note the presence of the round beads that were inserted to identify the transplant site.

The five day *in vitro* studies suggested that proliferation of epithelial cells in the ICCs may ultimately be a source for β-cells. However, there was no observed increase in the number of β-cells present in response to SDF-1α treatment in the time frame of the *in vitro* assay as measured by insulin content. RT-PCR studies also indicated that inhibition of CXCR4 *in vitro* did not alter the expression of transcription factors involved in the maturation of β-cells. Therefore, we next wanted to provide an extended differentiation period *in vivo* in the absence of SDF-1α for the ICCs. Both, control and AMD3100 treated ICCs were transplanted under the kidney capsule of immunodeficient mice. Post transplantation, mice were injected intraperitoneally with saline or AMD 3100 (5 µg/g) every other day for two weeks. Starting at eight weeks after transplantation, human C-peptide in serum from the mice was assayed. Mice that received control ICCs began to release human C-peptide after approximately four months, while mice treated with the CXCR4 inhibitor did not produce any human C-peptide (Fig. 8). The grafts of control mice showed extensive human insulin and glucagon positive cells (Fig. 9A and B), but the AMD3100 treated animals did not generate any insulin or glucagon staining at the transplant site (Fig. 9C). Beads grafted with the ICCs under the kidney capsule allowed us to definitively identify the transplant site in the absence of hormone positive cells (Fig. 9C). These studies underscore the importance of the SDF-1α/CXCR4 signaling axis in the development of insulin producing cells.

### The Effect of SDF-1α Stimulation on Proliferation of CFPAC-1s

**Figure 10:**
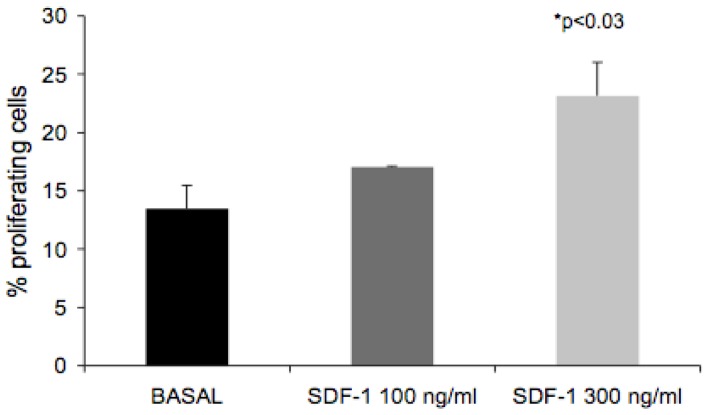
SDF-1 stimulates proliferation in CFPAC-1 cells. CFPAC-1 cells were seeded in 12 well plates and grown to 50–70% confluence. Following 24 hr serum starvation, cells were stimulated with SDF-1α (100 ng/ml or 300 ng/ml) for 16 hrs. BrdU was added 4 hrs before fixation. BrdU incorporation was determined as described under Materials and Methods. BrdU incorporation was stimulated 26.6% and 71.6% by 100 ng/ml and 300 ng/ml SDF-1α, respectively. Each bar represents the average of three experiments (mean ± SEM); P<0.03 by analysis of variance.

Treatment of ICCs *in vitro* showed that SDF-1α stimulates proliferation of epithelial cells in ICCs. We next addressed the mechanistic aspects of CXCR4 function. Considering that ICCs contain a heterogeneous cell population, we used the pancreatic ductal carcinoma cell line, CFPAC-1, that expresses CXCR4 (data not shown) and shares adhesion and migration characteristics with those of ICCs [27]. Initially, we addressed proliferation of CFPAC-1 cells following stimulation with SDF-1α for 24 hrs. Under basal conditions, 13% of the cells were proliferating. Treatment with SDF-1α increased proliferation in a dose dependent manner (Fig. 10). At 100 ng/ml, SDF-1α increased proliferation by 26.6%, while 300 ng/ml of SDF-1α augmented proliferation by 71.6%.

### The Effect of Inhibitors of PI 3-kinase, MAPK, PLC, and PKA Pathways on Basal and SDF-1α Stimulated Proliferation in CFPAC-1 Cells

**Figure 11:**
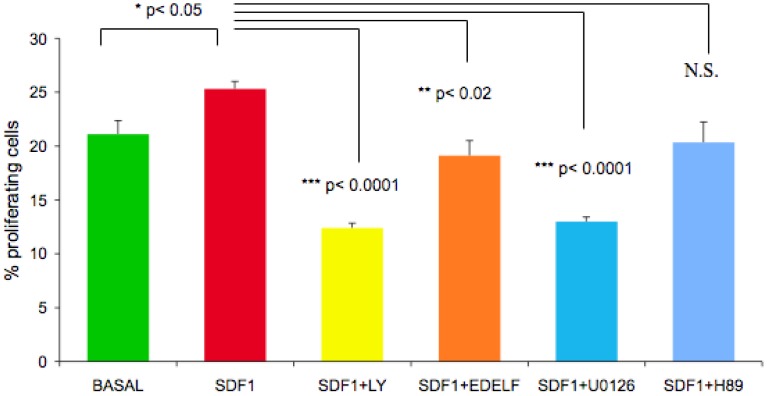
The effect of the inhibitors of PI 3-kinase, MAPK, PLC, and PKA on SDF-1α stimulated proliferation in CFPAC-1 cells. CFPAC-1 cells were seeded in 12 well plates and grown to 50–70% confluence. Following 24 hr serum starvation, cells were pre-treated with LY294002 (30 µM), U0126 (30 µM), Edelfosine (10 µM), or H89 (10 µM) for 30 minutes and stimulated with SDF-1α for 16 hrs. BrdU was added 4 hrs before fixation. BrdU incorporation was determined as described under Materials and Methods. (*, P<0.05 SDF-1 versus basal); (**, p<0.02 SDF-1/Edelfosine versus SDF-1 alone); (***, p<0.0001 SDF-1/LY294002 and SDF-1/U0216 versus SDF-1 alone) by Student’s t-test. N.S.  =  not significant by Student’s t-test.

CFPAC-1 cells were treated with a panel of pharmacological inhibitors to identify which signaling pathways downstream of CXCR4 were involved in proliferation (Fig. 11). Inhibition of the PI 3-kinase with LY294002 or MAPK with U0126 inhibited SDF-1α stimulated proliferation by 50% and 48% respectively. The PLC inhibitor, edelfosine, and the PKA inhibitor H89 inhibited SDF-1α stimulated proliferation to a lesser extent (<20%), while IBMX/forskolin, and JNK inhibitor II had no effect (data not shown).

### Effect of SDF-1α on Apoptosis in CFPAC-1 Cells

**Figure 12:**
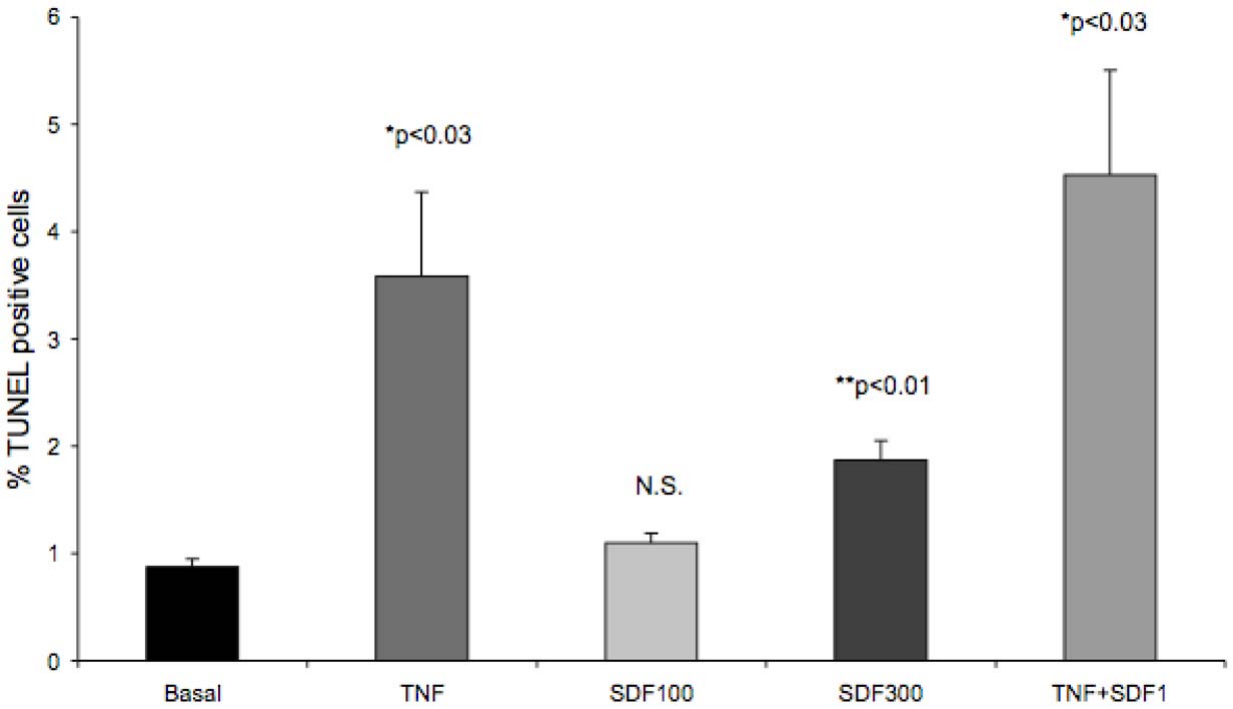
The cytokine cocktail TNFα, IL1β and IFNγ and SDF-1α (300 ng/ml) stimulate CFPAC-1 apoptosis. CFPAC-1 cells were seeded in 12 well plates and grown to 50–70% confluence. Following 24 hr serum starvation, cells were stimulated with either the cytokine cocktail (TNF) consisting of IL-1β (2 ng/ml), IFN-γ (100 ng/ml) and TNF-α (100 ng/ml), or SDF-1α at 100 ng/ml, 300 ng/ml, or the TNF cocktail in combination with SDF-1α 100/ng/ml for 24 hrs. The cells were fixed at the end of the incubation apoptosis was quantitated using the TUNEL method as described in the Materials and Methods. The number of TUNEL positive nuclei was expressed as the percentage of the total number cells counted in the acquired images. Data is expressed as mean ± SEM. (*, p<0.03 versus basal); (**, p<0.01 versus basal) by Student’s t-test. N.S.  =  not significant by Student’s t-test.

Over the course of the proliferation studies, we noticed that SDF-1α induced cell death. To explore this, we compared SDF-1α mediated apoptosis in CFPAC-1 cells to a combination of TNFα, IL1β and IFNγ that has been shown to cause apoptosis in islets [28]. This approach also allowed us to determine whether SDF-1α can counteract or synergize with the apoptotic effect of the cytokines specified above. In CFPAC-1 cells, a TNFα, IL1β and IFNγ cocktail induced apoptosis four-fold as measured by the percentage of TUNEL positive cells (Fig. 12). At low SDF-1α concentrations (100 ng/ml) apoptosis was unchanged; however, at high SDF-1α levels (300 ng/ml) an approximately two-fold increase in TUNEL positive cells was observed. The TNF cocktail in combination with SDF-1α (100 ng/ml) increased apoptosis approximately 5 fold. This increase was not statistically significant compared to TNF cocktail alone. Furthermore, combining the cytokine cocktail with SDF-1α at 300 ng/ml caused excessive cell death (data not shown).

### SDF-1α Stimulates Akt Phosphorylation in the Cell Line CFPAC-1s

**Figure 13:**
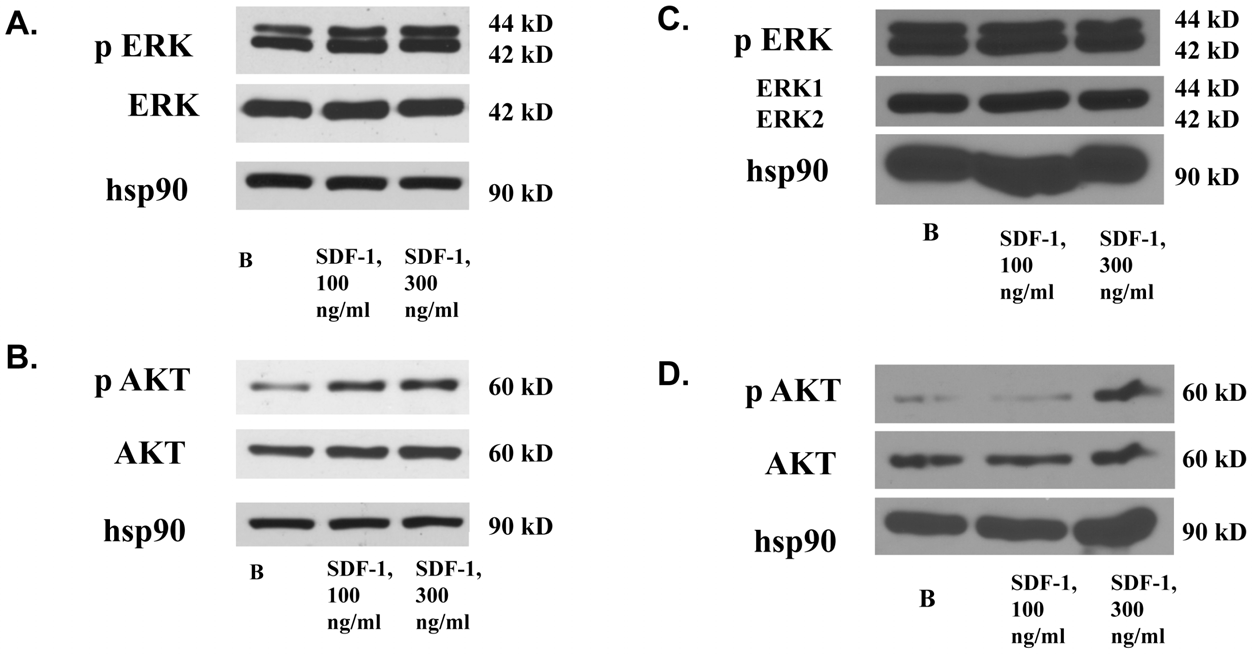
SDF-1α stimulates Akt but not MAPK phosphorylation in CFPAC-1 cells and fetal ICCs. Following 48 hr serum starvation, CFPAC-1 cells were stimulated with 100 ng/ml or 300 ng/ml human recombinant SDF-1α for 10 min at 37°C. Whole cell lystes were analyzed by western blot, using antibodies raised against dually phosphorylated phospho-MAPK(ERK1/ERK2)(A) or phospho-Akt (Ser473)(B). Following overnight serum starvation, ICCs from human fetal pancreas of 15 weeks gestation were stimulated with 100 ng/ml or 300 ng/ml SDF-1α for 10 min at 37°C. Whole cell lystes were analyzed by western blot, using an antibody raised against phospho-MAPK(C) or phospho-Akt (Ser473) (D). All blots were stripped and reblotted with antibodies to total Akt, Erk, and Hsp90 sequentially to confirm equal loading.

Inhibitor studies underscored the importance of PI 3-kinase and MAPK signaling pathway activation in SDF-1α-mediated proliferation. At least two distinct processes could be responsible for CFPAC-1 proliferation: decreased apoptosis through activation of PI 3-kinase signaling or increased proliferation through activation of MAPK. To determine the relative input of these downstream signaling pathways in CFPAC-1 proliferation, we next assessed the SDF-1α stimulated phosphorylation of MAPK and Akt *in vitro*. MAPK was phosphorylated in serum starved CFPAC-1 cells; addition of SDF-1α did not further stimulate MAPK phosphorylation significantly in at least four independent experiments (Fig. 13A). However, Akt was robustly phosphorylated at serine 473 in response to SDF-1α (100 ng/ml and 300 ng/ml) (Fig. 13B). To assess whether the signaling mechanisms activated by SDF-1α in the CFPAC-1s reflect those in the combination of epithelial, islet progenitor, and β-cells in ICCs derived from fetal pancreas, we performed parallel experiments in ICCs (from a 15.7 wk gestation human fetus) grown in suspension culture for four days. Consistent with the CFPAC-1 model system, basal MAPK phosphorylation was elevated despite serum starvation overnight and persisted at high levels in the presence of SDF-1α (Fig. 13C). SDF-1α stimulation of the ICCs resulted in the increased Akt phosphorylation at serine 473 (Fig. 13D). Taken together, our observations are consistent with a SDF-1α mediated effect on proliferation in fetal endocrine pancreas through Akt signaling.

## Discussion

Here we demonstrate that both the heterotrimeric G-protein coupled receptor CXCR4 and its ligand SDF-1α are expressed in the human fetal pancreas. CXCR4 co-localizes with Ngn3 positive cells in early gestation, and gradually becomes restricted to cells expressing insulin, but not glucagon. SDF-1α expression, on the other hand, does not overlap with hormone positive cells; rather its expression appears to be restricted to ductal structures in the pancreatic architecture. Treatment of human fetal ICCs with SDF-1α had no effect on insulin content, but it led to increased cell proliferation. Attenuation of CXCR4 activity by treatment with the inhibitor AMD3100 had no effect on expression of β-cell specific genes *in vitro* after 5 days. However, after transplantion of AMD3100 treated ICCs into nude mice, there was a profound inhibition of β-cell genesis. The survival and proliferative signals could be directly modulated downstream of CXCR4 by Akt in the cell line CFPAC-1. These results provide the first example of CXCR4 mediated signaling in the modulation of human β-cell growth and proliferation.

Expression of CXCR4 is a well-established cell surface marker for human embryonic stem cells lineage restricted to definitive endoderm [22], [29], but expression is subsequently diminished rapidly after this stage [29]. The co-localization of CXCR4 positive cells with ngn3, a transcription factor expressed in cells destined to become pancreatic endocrine cells indicates that CXCR4 expression is important in the further lineage restriction from endocrine progenitors to mature endocrine cells.

Important clues about the role of the CXCR4/SDF-1α during the transition from definitive endoderm to an endocrine precursor cell expressing the pancreatic precursor transcription factor PDX1 have recently been described by Katsumoto et al., who found that this signaling pathway plays an important role in establishing the fate of pancreatic progenitors in chick embryos [30]. These authors observed that overexpression of SDF-1α attracted cells expressing Lmo2, which subsequently induced *Pdx1*-expressing pancreatic progenitors and enhanced differentiation into insulin-expressing cells. Similar to our studies, treatment of cells with AMD3100 reduced pancreatic bud formation and treatment of cells with SDF-1α resulted in augmented cell proliferation.

SDF-1α stimulated proliferation has been documented in CD34(+) hematopoietic progenitors [31], [32] cerebellar granule cells [31] and astrocytes [32] from neonatal rodents. Conditional inactivation of CXCR4 in osteoprecursors has been shown to result in reduced postnatal bone formation, which was partially due to decreased osteoblast proliferation [33]. Our previous study using the IFNγ mouse, which displays regeneration in the ducts of the adult pancreas showed that inhibition of CXCR4 function by *in vivo* CXCR4 antibody administration inhibited ductal cell proliferation. *In vivo* the SDF-1α/CXCR4 axis can exert its effects at multiple levels [34], including adherance to the extracellular matrix, migration, further proliferation, and differentiation in response to endogenous growth factors. In a previous study, we reported that parenterally administered Exendin-4, a long-lasting analogue of Glucagon-Like-Peptide-1 (GLP-1), induces maturation of ICCs transplanted into nude mice [24]. Liu et al have recently postulated a connection between SDF-1α secretion and GLP-1 production in injured islets [24], [35]. According to this recent report, injury to the islets results in SDF-1α release. In response to SDF-1α GLP-1 is produced by the alpha cells and feeds back to induce the growth and survival of β-cells. In the present study, the SDF-1α stimulated proliferation in ICCs derived from the human embryonic pancreas was comparable to the effect of HGF, a growth factor and primary component of the mesenchyme induced β-cell growth in human fetal ICCs [26]. The ability of SDF-1α to induce the proliferation of pancreatic epithelial cells may provide an increased number of precursors for subsequent differentiation into β-cells *in vivo*.

*In vitro* studies demonstrated a clear capacity of SDF-1α to enhance replication of human fetal ICCs (Fig. 6C); however, RT-PCR analysis revealed no significant increase in endocrine cell markers, including PDX-1, Ngn3, Pax-4, Nkx 6.1, or insulin (Fig. 5 and 7). One possible explanation is that the proliferating epithelial cells are not of endocrine origin. However, the results from the transplant studies suggest an alternate explanation (Fig. 8 and 9). Long-term treatment of fetal ICCs with the CXCR4 specific inhibitor AMD3100 blocks both cell growth and C-peptide release, suggesting that the SDF-1α/CXCR4 signaling axis is essential for cells to survive and develop into insulin secreting cells. The latter observation suggests that a temporal aspect of CXCR4 mediated signaling is critical for development *in vivo* after cell transplantation that might not be observed in a short *in vitro* experiment.

To examine the dynamics of CXCR4/SDF-1α expression in development, signaling related to proliferation and apoptosis was explored in a cell line to avoid the effects of heterogeneity in a fetal ICC population. CXCR4 is a Giα-coupled heterotrimeric heptahelical transmembrane protein with well-defined signaling outputs [36]. Pharmacological manipulation of these signaling events allowed us to define pathways important for the observed effects. Both edelfosine (a PLC inhibitor) and H89 (a PKA inhibitor) failed to alter the effects of SDF-1α treatment. However, inhibition of PI 3-kinase or canonical MAP kinase signaling attenuated proliferation (Fig. 11 and data not shown), suggesting signaling from CXCR4 through these two pathways was essential for cell function. These results are consistent with our findings in the mouse, where we demonstrated that SDF-1α stimulates the phosphorylation of Akt and mitogen-activated protein (MAP) kinase in pancreatic duct cells [14].

In mouse islets, SDF-1α expression was restricted to cells surrounding the ducts and the microvasculature both around the ducts and in the islets [20]. SDF-1α expression was also observed in the β-cells of the pancreas in neonatal mice up to 21 days of age, after which SDF-1α was no longer expressed in the β-cells [35]. We have also observed SDF-1α expression the ducts and cells immediately surrounding the ducts in the human adult pancreas (Fig. 4B) with no apparent expression in the islets, suggesting similarities between the two systems. Further characterization of the SDF-1α expression in the human pancreas will be addressed in future studies.

In the stepwise differentiation of human embryonic stem cells the expression of CXCR4 has been established as a marker of definitive endoderm [22]. Habener et al have reported that mice expressing SDF-1α under the control of the insulin promoter (RIP-SDF-1α mice) are somewhat protected against STZ-induced diabetes. Their studies also showed SDF-1α induced promotion of β-cell survival by Akt activation [21]. Subsequent studies by the same group have suggested that the activation of the WNT pathway by SDF-1α may be part of the mechanism that promotes β-cell survival [37]. Therefore, the stimulation of Akt in the absence of an anti-apoptotic effect is intriguing. Taken together with the lack of an effect on MAPK, the SDF-1α effect on proliferation in this context appears to be Akt dependent. SDF-1α stimulates proliferation in human cortical neural progenitor cells derived from human fetal brain tissue. This proliferative effect has been shown to be dependent on Akt phosphorylation [38]. The Akt pathway, the primary mediator of PI 3-kinase signaling, has also been shown to regulate the proliferation of β-cells, an effect that involves GSK3 and Cyclins D1 and D2 [39], [40], [41].

Cell growth during embryonic development and disease is a tightly orchestrated process that ensures the proliferation of certain cells while allowing the apoptosis of others [42], [43]. CXCR4 has been has been shown to be involved in the stimulation of apoptosis by HIV in both T cells and neurons [44], [45], [46], [47], [48]. SDF-1α has also been shown to directly stimulate apoptosis in neural cells [49]. Frequently, SDF-1α stimulation is associated with enhanced survival as reported in hematopoietic progenitor cells [50], fetal thymus [51], [52] and in bone marrow myelopoiesis [52]. Previous studies have shown that SDF-1α has a pro-survival effect in mouse pancreas, MIN-6 cells, and INS-1 cells [14], [21], [27]. Our observation of SDF-1α stimulation of apoptosis in CFPAC-1 cells was unexpected. It is conceivable that the stimulatory effect of SDF-1α at high concentrations (300 ng/ml) may be representative of what happens to ICCs at a particular stage of development. The *in vivo* relevance of this apoptotic effect requires further study.

Taken in context, our results suggest that the presence of the CXCR4/SDF-1α axis in the early pancreas is important for lineage restriction to endocrine cells, while its persistence in the adult may indicate either a potential for regeneration or an active role in proliferation, survival and maintenance of β-cells.

## Materials and Methods

### Human Tissue

#### Human tissue

Human fetal pancreata used in this study (10 to 17 weeks gestation) were provided by Birth Defects Research Laboratory, University of Washington (Seattle, Wash., USA.) Informed consent for tissue donation, storage, and use of the samples was obtained from the donors by the center. The protocol #081237XT consent statement was in writing. Furthermore, The University of California, San Diego Human Research Protections Program approved the whole study (Protocol #081237XT).

The University of California, San Diego Human Research Protections Program approved the use of human adult tissue for the experiments (Protocol #07943XT). The protocol #071943XT consent statement was in writing. Human adult pancreas biopsies (block) were collected and processed at The University of California, San Diego. The Director of the Human Research Protections Program certified that Project #120578XX (Mechanisms of Pancreatic Development-2) is exempt from IRB approval under 45 CFR 46.101(b)), category 4: Research involving the collection or study of existing data, documents, records, pathological specimens, or diagnostic specimens, if these sources are publically available or if the information is recorded by the investigator in such a manner that subjects cannot be identified directly or through identifiers linked to the subjects. The samples were anonymized.

The University of California, San Diego Institutional Animal Care and Use Committee approved the use of athymic mice for the transplant experiments (Protocol #S00175M). The protocol #S00175 consent statement was in writing.

### Preparation and Treatment of Fetal Pancreatic Islet-like Cell Clusters (ICCs)

Fetal pancreata were processed as described previously (Beattie, 1994). Tissue was minced and digested with collagenase Type XI (Sigma, St. Louis, Mo., USA) and allowed to form islet-like clusters in suspension in RPMI-1640 containing 10% human AB serum (Cellgro, Mediatech, Manassas, VA.). Following the formation of ICCs, the clusters were treated with HGF (10 ng/ml) (a generous gift by Genentech, San Francisco, CA.), SDF-1α (100 ng/ml) (PeproTech Inc. Rocky Hill, NJ.) or AMD 3100 (1 µg/ml) (Sigma, St. Louis, MO.) for 5 days with a medium changes every two days. At the end of the *in vitro* treatment for some experiments the ICCs were processed for insulin extraction and DNA quantitation as described previously [53]. Some ICCs were fixed in 4% paraformaldehyde, embedded first in agarose and then in paraffin for histological analysis.

### Transplantation Experiments

Nu/nu athymic mice were obtained from the in-house breeding colony at the Animal Care Program, University of California, San Diego. 500 to 1000 islet-like cell clusters that were treated *in vitro* with either phosphate buffered saline or AMD3100 were placed under the kidney capsule of five athymic mice each for control and treatment groups using a positive displacement pipette as described previously [54]. The transplanted mice were treated with vehicle (saline) or AMD3100 (5 mg/kg) intraperitoneally every other day for two weeks. Eight weeks after transplantation, fasted animals were given 3 g/kg glucose intraperitoneally, and after 30 min; blood samples were taken for the assay of circulating human C-peptide with a ELISA kit (Mercodia, Inc.) that is specific for human C-peptide, with no cross-reactivity with mouse C-peptide. The glucose stimulations were repeated every four weeks until circulating human C-peptide was detected. At four to five months, the mice were sacrificed and serial sections of the kidneys bearing grafts were examined histologically for insulin and glucagon cells as described below. Human C-peptide levels in serum of transplanted mice were measured with enzyme-linked immunoabsorbent assay (ELISA) kits (Mercodia, Inc.).

### Immunofluorescence

Human fetal or adult pancreata were fixed in 4% paraformaldehyde and embedded in paraffin. Paraffin-embedded tissue was cut into 4-µm sections and stained with goat polyclonal anti-CXCR4 antibody raised against the NH_2_ terminal extracellular domain of the human CXCR4 receptor (Caprologics, Inc. Gibertville, MA.), mouse monoclonal anti-SDF-1α antibody raised against recombinant human SDF-1α (R&D Systems, Inc.), sheep polyclonal anti-insulin antibody (The Binding Site, Inc. San Diego, CA.), mouse monoclonal anti-insulin antibody (Sigma, St. Louis, MO.), and mouse monoclonal anti-glucagon (Sigma, St. Louis, MO.). The visualization of CXCR4 required the use of biotinylated goat secondary (Jackson Immunoresearch Labs, Inc.) followed by Streptavidin conjugated Alexa 488 or 536 (Invitrogen, Molecular Probes Inc.). Following the Alexa Flour incubation, the sections were placed in mounting medium (Fluorogel with Tris Buffer (Electron Microscopy Sciences, Hartfield, PA.). For the Ngn3 and CXCR4 co-expression experiments, human fetal pancreas was cryopreserved in optimal cutting temperature (Tissue-Tek, Sakura Finetek USA, Torrance, CA.) and 4 µm sections were cut for staining. Sheep anti-human Ngn3 antibody raised against recombinant human Ngn3 (R&D Systems, Inc.) and rabbit anti-CXCR4 antibody raised against the N-terminal amino acids 1–14 of the human CXCR4 receptor (Abcam, Cambridge, MA.) were used on the frozen tissue. The ICCs treated *in vitro* with HGF, SDF-1α or AMD3100 were stained with rabbit anti human Ki67 antibody (Neomarkers, Fremont, CA.) and mouse anti-human (large spectrum) cytokeratin (Immunotech, Coulter, Cedex, France).

### Assessment of SDF-1α Effect on Proliferation

The CFPAC-1 cells (ATCC) were grown in monolayer culture in 12 well plates to 50–70% confluency using Iscove’s Modified Dulbecco’s medium containing 10% fetal bovine serum. The cells were then serum starved for 24 hrs and stimulated with SDF-1α (100 or 300 ng/ml) for 12 hrs, BrdU wad added and the cells were incubated another 4 hrs and then fixed for 20 min in 4% paraformaldehyde and stained with rat monoclonal anti-BrdU antibody (Abcam, Cambridge, MA.) followed by anti-rat Alexa 488 (Invitrogen, Molecular Probes, Inc.) secondary antibody. DRAQ5 (Cell Signaling Technology) was used to visualize nuclei. Each treatment was done in triplicate and eight images per well were acquired on a Zeiss Axiovert microscope (Carl Zeiss Microimaging, Inc., Thornwood, NY.) using a MicroMax digital camera (Roper-Princeton Instruments, Acton, MA.) controlled by MetaFluor software (Universal Imaging, Corp., Sunnyvale, CA.). The percentage of proliferating cells was calculated by expressing the number of BrdU positive nuclei divided by the total number of nuclei in the captured images. LY294002, U0126, Edelfosine, H89, IBMX, Forskolin, and JNK inhibitor II were obtained from EMD Biosciences Inc. (San Diego, CA.) The inhibitors were added following the 24 hr. serum starvation 30 min prior to stimulation with SDF-1 α.

### Real-time-PCR

Total RNA was isolated using an RNeasy PlusMini Kit 50 Qiagen, (Valencia, CA) and cDNA was synthesized using High Capacity cDNA Reverse Transcription Kit (Applied Biosystems, Foster City, CA). Quantitative PCR was performed on a StepOne Plus thermocycler (Life Technologies) with SYBR green mastermix or Taqman mastermix (Life Technologies). mRNA Ct values were normalized to either Cyclophilin G or Cyclophilin A. The PCR primers (Invitrogen, San Diego, CA) and TaqManFAM probes (Applied Biosystems) used are listed in Table S1.

### Assessment of SDF-1α Effect on Apoptosis

The CFPAC-1 cells grown on glass coverslips to 50–70% confluency 12 well dishes were serum starved for 24 hrs and stimulated with a cytokine cocktail comprising IL-1β (2 ng/ml), IFN-γ (100 ng/ml) and TNF-α (100 ng/ml) or SDF-1α (100 or 300 ng/ml) or a combination of the cytokine cocktail and SDF-1α for 24 hrs. Recombinant human IL-1β, IFN-γ and TNF-α were obtained from R&D Systems, Inc. (Minneapolis, MN.) The concentrations of the cytokines used were based on their apoptotic effect on human islets [55]. The cells were fixed with 4% paraformaldehyde at the end of the incubations and TUNEL staining was performed using the In Situ Cell Death Detection Kit, POD kit (Roche, Indianapolis, IN.) according to manufacturer’s instructions. Eight images were captured from each well and each treatment was performed in triplicate.

### Assessment of SDF-1α Stimulation of MAPK and Akt Phosphorylation

CFPAC-1 cells were serum starved for 48 hrs and stimulated with 100 or 300 ng/ml human SDF-1α (PeproTech Inc. Rocky Hill, NJ.) for ten minutes at 37°C. Cells were lysed with RIPA buffer containing 20 mmol/liter Tris, pH 7.5, 1 mmol/liter EDTA, 140 mmol/liter NaCl, 1% NP-40, 1 mmol/liter orthovanadate, 1 mmol/liter PMSF, 2 mmol/l sodium pyrophosphate, 25 mmole/l α-glycerophosphate, 10 mmol/l sodium fluoride, 10 µg/ml each of aprotinin, leupeptin, and pepstatin. Equal amounts of protein were subjected to western blot analysis. Rabbit polyclonal antibodies to dually phosphorylated phospho-MAPK (Thr202/Tyr204) and to phospho-Akt (Ser473) were used in immunodetection (Cell Signaling Technology). The membranes were stripped and reblotted with mouse monoclonal Erk and rabbit polyclonal Akt antibodies (Cell Signaling Technology) to visualize total Erk and Akt expression. Finally the membranes were stripped and reblotted with a mouse monoclonal Hsp90 antibody to confirm equal protein loading (BD Transduction Laboratories, San Diego, CA.).

## Supporting Information

Table S1 Primers and probes used in this study.
(DOCX)
