# Supplementary material for: The SDF-1α/CXCR4 Axis is Required for Proliferation and Maturation of Human Fetal Pancreatic Endocrine Progenitor Cells
Source: PLoS One. 2012 Jun 22;7(6):e38721. doi: 10.1371/journal.pone.0038721 (PMC3382144; doi:10.1371/journal.pone.0038721)
Supplement: Supplementary file 1 [file pone.0038721.s001.docx]

Table S1: Primers and probes used in this study

Gene name	Abbreviation	Primer sequence or Taqman Assay ID#
Cyclophilin G (housekeeping gene)	CycG F<br>CycG R	CTTGTCAATGGCCAACAGAGG<br>GCCCATCTAAATGAGGAGTTGGT
Cyclophilin A (housekeeping gene)	CycA	ABI Taqman assay# Hs99999904_m1
Chemokine (C-X-C motif) receptor 4	CXCR4 F<br>CXCR4 R	CCGTGGCAAACTGGTACTTT<br>TTCCTTGGCCTCTGACTGTT
Glucagon	Glu	ABI Taqman assay# Hs01031536 m1
Insulin	Ins	ABI Taqman assay# Hs00366773 m1
V-maf musculoaponeurotic fibrosarcoma oncogene homolog A (avian)	Maf A	ABI Taqman assay# Hs01651425_s1
Neurogenin 3	Ngn3	ABI Taqman assay# Hs00360700_g1
NK6 homeobox 1	Nkx6.1	ABI Taqman assay# Hs00232355_m1
Paired Box 4	Pax4	ABI Taqman assay# Hs00173014_m1
Insulin promoter factor 1	Pdx1	ABI Taqman assay# Hs 00426216_m1
Stromal cell derived-factor-1	SDF1 F<br>SDF1 R	GATTGTAGCCCGGCTGAAGA<br>TTCGGGTCAATGCACACTTGT
